# Using deep reinforcement learning to speed up collective cell migration

**DOI:** 10.1186/s12859-019-3126-5

**Published:** 2019-11-25

**Authors:** Hanxu Hou, Tian Gan, Yaodong Yang, Xianglei Zhu, Sen Liu, Weiming Guo, Jianye Hao

**Affiliations:** 1 School of Electrical Engineering & Intelligentization, Dongguan University of Technology, No.1 University Road, DongGuan, 523808 China; 2 College of Intelligence and Computing, TianJin University, No.135 Yaguan Road, TianJin, 300350 China; 3 Automotive Data Center, CATARC, No.69 Xianfeng Road, TianJin, 300300 China

**Keywords:** Collective migration, Leader-follower mechanism, Deep reinforcement learning

## Abstract

**Background:**

Collective cell migration is a significant and complex phenomenon that affects many basic biological processes. The coordination between leader cell and follower cell affects the rate of collective cell migration. However, there are still very few papers on the impacts of the stimulus signal released by the leader on the follower. Tracking cell movement using 3D time-lapse microscopy images provides an unprecedented opportunity to systematically study and analyze collective cell migration.

**Results:**

Recently, deep reinforcement learning algorithms have become very popular. In our paper, we also use this method to train the number of cells and control signals. By experimenting with single-follower cell and multi-follower cells, it is concluded that the number of stimulation signals is proportional to the rate of collective movement of the cells. Such research provides a more diverse approach and approach to studying biological problems.

**Conclusion:**

Traditional research methods are always based on real-life scenarios, but as the number of cells grows exponentially, the research process is too time consuming. Agent-based modeling is a robust framework that approximates cells to isotropic, elastic, and sticky objects. In this paper, an agent-based modeling framework is used to establish a simulation platform for simulating collective cell migration. The goal of the platform is to build a biomimetic environment to demonstrate the importance of stimuli between the leading and following cells.

## Background

Cell migration is a complex and highly dynamic phenomenon which is primarily driven by the action network beneath the cell membranes and is essential to a variety of biological processes such as the development of an organism, wound healing, cancer metastasis and immune response[1]. For example, during morphogenesis, there is a targeted movement of dividing cells to form tissues and organs. For wound healing to occur, cells such as neutrophils (white blood cells) and macrophages (cells that ingest bacteria) move to the wound site to kill the mocroorganisms that cause infection, and fibroblasts (connective tissue cells) move there to remodel damaged structures [1–3]. Active directional collective cell migration is basic mechanism of cell migration that enables the coordinated movement of groups of cells that remain connected via cell-cell junctions during morphogenesis, wound repair and cancer invasion [4, 5]. The guide of collective migration often involves the coordination between two functionally distinct populations, leader and follower cells. Leader cells localize at the front of a moving group, where they receive the guidance signals and instruct, with cell-cell junctions at their rear, follower cells into directional migration through chemical and/or mechanical signaling [6, 7].

There are massive recordings, which can efficiently track large numbers of migrating cells in 4D movies of morphogenesis model, provide a unique opportunity for cellular-level behavior recognition as well as simulation-based hypothesis testing [8]. Recently development in cutting-edge live microscopy and image analysis provide an unprecedented opportunity to systematically investigate cell migration and simulate leader-follower cellular behaviors movement extended period of time [9, 10].

Agent-based modeling is a powerful approach that approximates cells as isotropic, elastic and adhesive objects. Cell migration is modeled by an equation of motion for each cell [11]. In which all cells and environment parameters can be independently varied which facilitates species specific simulation and allows for detailed analyses of growth dynamics and links between cellular and multi-cellular phenotypes [12–14]. Therefore the framework of agent-based modeling is used in our present work to simulate collective cell migration driven by leader-follower mechanism.

Reinforcement learning dates back to the early days of cybernetics and work in statistics, psychology, neuroscience, and computer science [15, 16]. An agent that must learn behavior through trial-and-error interactions with a dynamic environment by obtaining reward and punishment without needing to specify how the task is to be achieved. So far, reinforcement learning had some successes in many aspects [17, 18]. But previous approaches lacked scalability and were inherently limited to fairly low-dimensional problems. These limitations exist because reinforcement learning algorithms share the same complexity issues as other algorithms: memory complexity, computational complexity, and in the case of machine learning algorithms, sample complexity [19]. What we have witnessed in recent years—the rise of deep learning, relying on the powerful function approximation and representation learning properties of deep neural networks—has provided us with new tools to overcoming these problems [20, 21].

In this paper, we not only consider the case of a single agent, but also consider the situation of multi-agents. A multiagent system [22] can be defined as a group of autonomous, interacting entities sharing a common environment, which they perceive with sensors and upon which they act with actuators [23]. Unlike the previous single-agent reinforcement learning, multi-agent promotes its own strategy while also considering the cumulative rewards of other agents. Furthermore, most of the times each learning agent must keep track of the other learning (and therefore, nonstationary) agents [24]. Only then will it be able to coordinate its behavior with theirs, such that a coherent joint behavior results [25]. The nonstationarity also invalidates the convergence properties of most single-agent RL algorithms [26]. In addition, the scalability of algorithms to realistic problem sizes, already problematic in single-agent RL, is an even greater cause for concern in multiagent reinforcement learning (MARL) [27]. The second experiment of this article is based on multiagent.

This paper proposes a new research method to study the relationship between this stimulus signal(including chemical and mechanical signaling) and collective cell movement by using deep reinforcement learning in an agent-based model to control the stimulation signals released to the leading cells following the cell. In our current work, an individual cell is modeled as an agent, where modeling includes the environment in which the cell is located, the size of the cell itself, the rules of the biological environment that the cell needs to follow, and the relative location of the cell neighbor cells. Of course, the process of cell migration generally includes two aspects, one is its own mode of movement as a single individual cell, and the other is the mode of movement of the collective cells of the group in which the cells are located. The experiments herein focus on the second aspect described above.

The writing framework of this article is roughly as follows: firstly, the background and significance of the migration of collective cell migration are highlighted. In addition, in this part, the methods of this paper are also defined, including the model, construction and setting of specific algorithms, etc. Then, in the second section, the details of the experiment and the results are elaborated. Finally, in the third quarter, we propose the direction that is worthwhile to continue research in the future.

### Collective cell migration

First, a brief introduction to collective cell migration can be defined that cells move together, making contact at least some of the time, and if they affect one another while migrating.

For all animals, cell migration is an essential and highly regulated process [28]. In a system of collective cell migration, individual cell movement can be a part of the system with specific communication between them. Among cells, chemical and mechanical signals are considered as specific communication methods [29]. The collective migration of cells is affected by many specific factors. Among them, the influencing factors considered in this paper are specific biological signals and the inherent biological relationships between cells and cells, while ignoring other factors. Biological signal can also be understood as an alternating medium between biological cells. This medium helps guide, shape and ensure the final formation of new cell morphology. If there is no biological signal as a bridge, the movement of the cells will begin to control and begin to move randomly, which will not help to repair the biological function. This article does not describe how specific biological signals are produced and functioned in cells, primarily by simulating the importance of the presence of biological signals and how to control them to affect the migration of collective cells.

Many cells can move in distinct situations or at a specific developmental time which can places, shapes or repairs the tissue of which they are part [30]. Combined with the first single cell experiment in this article, we will study how the migration process of collective cells is implemented on a bionic platform.

### Leading-following mechanism

In the system of collective cell migration, there is a mechanism by which the leading cell interacts with the following cell. Many results may indicate that pulling forward by the migrating leader cell is a mechanical trigger for subsequent migration of follower cells [31, 32]. The so-called leading cells, that is, located in front of the collective cell migration, can play a guiding role for other cells. When cells begin to migrate collectively, the leading cells are induced by some trigger factors and simultaneously are activated to initiate departure from the epithelium but remains attached to adjacent follower cells that are also able to reorganize. At the same time, the following unit is simulated by the leader unit and begins to move to the leader unit. Without being known by the leading cells, collective cell migration loses its ability to move in a certain direction and becomes a random exploratory move.

What is said above is the main role of the leading cells in the collective migration, then how is the leading cell and the following cells established and how the connection is established. The bio-stimulation signal that leading the cell is communicated to the follower’s cells. By studying this communication relationship on the simulation platform, it will help future biologists to study biology on a bionic platform.

## Method

### Deep reinforcement learning

**Fig. 1:**
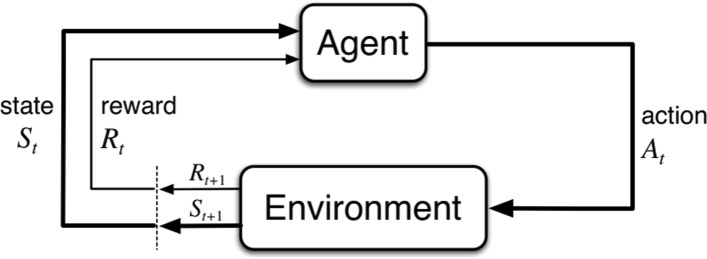
The reinforcement learning framework: leader cell or follower cell which are seen as the agent, continually interact with environment. The agent selects actions and the environment responds to these actions and presents new situations to the agent. The environment also gives rise to rewards, special numerical values that the agent seeks to maximize over time through its choice of actions

Reinforcement learning(RL) is learning what to do—how to map situations to actions—to maximize a numerical reward signal. The learner is not told which actions to take but instead must discover which actions the most rewards by trying them. In the most interesting and challenging cases, actions may affect not only the immediate reward but also the next situation and, through that, all subsequent rewards [33, 34]. The thing learner or decision maker interacts with, comprising everything outside the agent, is called the environment. An agent directly interacts with its environment without relying on exemplary supervision or complete models of the environment. RL can be defined as a tuple (*S*,*A*,*R*,*P*,*γ*). *S* represents the state space and *A* represents the action space. *R* represents the immediate reward function, $R: S \times A \rightarrow \mathbb {R}$. *P* represents the state transition dynamics, *S*×*A*×*S*→[0,1]. *γ*∈(0,1) called the discount rate, use to calculate the cumulative return. The whole reinforcement learning process is demonstrated in Fig. 1.

Although reinforcement learning has made great success in many domains, previous approaches lacked scalability and were inherently limited to fairly low-dimensional state spaces. Here we witnessed in the popular deep reinforcement learning which has been developed in recent years. The most important property of deep learning is that deep neural network can automatically find compact low-dimensional features of high-dimensional data. Deep learning also enables RL to resolves more intractable problems which is high-dimensional state and action space—with the use of deep learning algorithms within RL defining the field of "deep reinforcement learning"(DRL). In recent years, there are massive novel algorithms in the DRL field that can effectively solve large and complex problems, but in this paper, the Deep-Q-Network(DQN) is mainly used to research the collective cell migration and this specific algorithm introduction will be elaborated later.

### Simulation framework

*1) ABM platform:* ABM(Agent-based modeling) is an effective framework to simulate fundamental cells behaviors which contains cell fate, cell division, cell migration [35]. It transforms biological problems into mathematical models and computer models to track the complex processes of cell movement and cell migration. In the modeling process of agent-based modeling, it is necessary to make the shape of cell movement under the simulated scene as close as possible to the shape of cell movement under the real scene. Based on the AMB model, the environmental information obtained by using 3D image processing technology establishes a complete motion model for different states of cells at different times. In this model, the influence process of the stimulation signal is added. Among them, it will involve the frequency of the stimulation signal, the amount of the stimulation signal, and the like, and the change of the rate of collective migration under the influence of different factors. The source of the specific cell position is the data mentioned in the literature [35]. The relative positional relationship between cells and neighboring cells represents the environment in which the cells are located. These relative relationships are essential and affect many fundamental biological processes, including cell signaling, cell migration, and cell proliferation.

Among them, the cell movement is not at random but is subject to specific rules. As described in literature [35], in the deep reinforcement learning scenario, this rules that guide cell movements can be transformed to reward function as an evaluation of how well a cell moves during a certain period based on those mechanisms. In our present work, we mainly consider the following three rules(the setting of these rules’ rewards will be described later):

- *the Boundary Rule:* Cell can’t break through the eggshell. With a certain range of cell and eggshell, the closer a cell is to the eggshell, the higher the penalty it will receive from the environment. Therefore, the cell must learn to keep an appropriate distance with eggshell.
- *the Collision Rule:* Cell can’t excessively squeeze cells around it. When the distance between two cells is less than a certain range, they will receive a punishment from environment. Therefore, the cell must learn to keep an appropriate distance with cells around itself.
- *the Destination Rule:* Cell movement is always directional and usually chooses the optimal path to reach the target. For the leader-follower mechanism, leader cell seeks the optimal path to reach the target, meanwhile the follower cell behind leader cell track the trajectory of the leader cell to move.

The introduction of these rules will be used later when the rewards set in the DQN algorithm. However, this paper will not discuss in detail how these rules appear, mainly to contrast the effect between leader cell gives follower cell stimulating signals and leader cell does not give stimulating signals.

*2):Deep-Q-Network:* In this algorithms, an individual cell is seen as an agent and the position of the cell is regarded as the state—*S*. The relative positional relationship between cells and rules to be considered to be the environment. The direction in which each cell can move can be seen as action—*A*, a total of 8 actions. At each discrete time step *t*, the cell senses its environment state *S*_*t*_∈*S* from an embryo and selects an action *A*_*t*_∈*A*. The environment returns a numerical reward *R*_*t*_∈*R* to the cell as an evaluation of that action at that state. The reward includes three rules as mentioned in the previous section, for boundary rule and collision rule, once a threshold of distance is reached, a terminal condition is triggered and the process restarts. For the destination rule, when the cell is closer to the target, the environment gives the cell a greater reward, which in turn stimulates the cell to move toward the target.

The main algorithm of this paper is still based on the DQN algorithm of deep reinforcement learning that has been studied before, which is mainly inspired by the innovative significance of application. More research paradigms of artificial intelligence can be extended to the industry to help researchers in different fields. The network is trained with traditional Q-learning [36] and use deep convolutional neural network [37, 38] which is fed with cells’ state and outputs a value for each cell’s action to approximate the optimal action-value(as known as *Q*) function. The optimal action-value is defined as Eq. (1), where *π* is a policy mapping sequences to actions of cells (or distributions over actions). That is, in the environment, each individual cell is considered an agent. At each state the agent selects an action *a*_*t*_ at from eight legal actions, then it receives a reward *r*_*t*_ represent the immediate rewards. The agent’s goal is to maximize the total amount of reward it receives. This means maximizing not immediate reward, but cumulative reward in the long run. In other words, the goal of agent can be thought of as the maximization of the expected value of the cumulative sum of a received reward. If the sequence of rewards received after time step *t* is denoted *r*_*t*_, *r*_*t*+1_, *r*_*t*+2_, *r*_*t*+3_, …, then the return is the sum of the rewards: *r*_*t*_ + *r*_*t*+1_ + *r*_*t*+2_+…. The additional concept that we need is that of discount rate. The discount rate determines the present value of the future rewards: a rewards received *k* time steps in the future is worth only *γ*^(^*k*−1) time what it would be worth if it were received immediately. The agent select actions to maximize the expected discounted return: *r*_*t*_ + *γr*_*t*+1_ + *γ*^2^*r*_*t*+2_ + ….

Reinforcement learning is known to be unstable or even to diverge, the specific reasons for it can be found in the [37, 38]. Deep reinforcement learning combines the perception of deep learning with the decision-making ability of reinforcement learning. It can be directly controlled according to the input image. It is an artificial intelligence method that is closer to the human way of thinking. DQN can improve these two shortcomings, mainly including changing the data distribution and correlations between the action-values(*Q*) and the target values *r*+*γ* max*a*^′^*Q*(*s*,*a*).

1 $$ {\begin{aligned} Q^{*}(s,a) = \max_{\pi} \mathbb{E}\left[\left.r_{t} + \gamma r_{t+1} +\gamma^{2} r_{t+2}+\ldots\right|s_{t}\right.\\ \left.=s,a_{t}=a,\pi\right] \end{aligned}}  $$

DQN combines convolutional neural network with Q learning, and solves the problem of combining DL and RL by the following methods: (1)firstly, DQN used a biologically inspired mechanism termed experience replay— *D*=*e*_1_,*e*_2_,...,*e*_*N*_ that randomizes over the data, thereby removing correlations in the observation sequence of cells and smoothing over changes in the cells’ data distribution. In this algorithm, an approximate value function *Q*(*s*,*a*;*θ*_*i*_) is parameterized using the deep convolutional neural network, in which *θ*_*i*_ are the weights of the Q-network at iteration *i*. In our present work, we store the agent’s experience into replay buffer at each time-step *t* in a data *e*_*t*_=(*s*_*t*_,*a*_*t*_,*r*_*t*_,*s*_*t*+1_), and then randomly select samples for training at each time and use the fixed length representation of histories produced by a function *ϕ*. (2) secondly, DQN uses an iterative update that adjusts the action-values (*Q*) towards target value that is only periodically updated, thereby reducing correction with the target. In the target network, rather than updating the weight in the single neural network, the weight remains unchanged for all n iterations until they are updated with $\theta _{i}^{-}$ from online network.

Leader cell and follower cell are trained using DQN algorithm, and their update process can be achieved by minimizing the loss function *L* defined as Eq. (2). Then backpropagating the loss through the whole neural network to update *θ* by *θ*_*t*_+1=*θ*_*t*_−*α*∇*L*(*θ*_*t*_), where *α* is the learning rate. For leader cell and follower cell an *ε*-greedy strategy was implemented, which is the most of time agent always exploits current knowledge to maximize their immediate reward and meanwhile with small probability *ε* independently of the action-value estimates. This method can balance exploration and exploitation.

2 $$ {{}\begin{aligned} L(S_{t},A_{t}|\theta_{t}) \,=\, \left[R_{t} \,+\, \gamma \max_{a} Q(S_{t+1},A_{t+1}|\theta_{t}) \,-\, Q(S_{t},A_{t}|\theta_{t})\right]^{2} \end{aligned}}  $$

3 $$ {{}\begin{aligned} y_{j}=\left\{ \begin{array}{lcl} r_{j}&\text{for terminal }\phi_{j+1}\\ r_{j}+\gamma \max_{a}{Q^{*}(\phi(s_{t}),a;\theta)}&\text{for non-terminal }\phi_{j+1} \end{array}\right. \end{aligned}}  $$

The full algorithm of DQN, in the general case, is outlined in Algorithm 1.


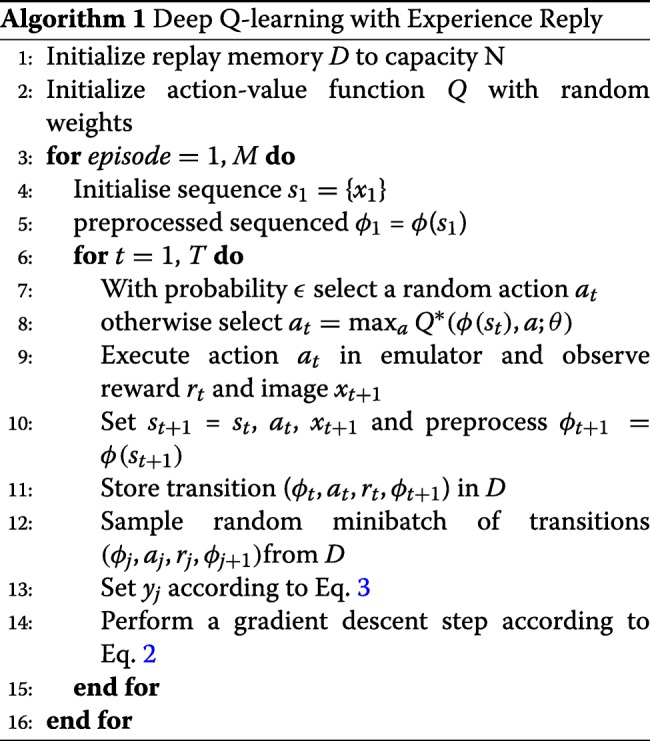


*3):Algorithm Setting:* Next, I will mainly introduce the work related to algorithm setting. During the experiment, it is necessary to ensure that the relative positional relationship between the cells and the cells is unchanged, and the appropriate reward function is used to indicate how the stimulating factors before the cells affect the migration of the collective cells.

- *Relative Position of Cells:* It is critical to ensure that the information entered is as similar as possible to the real environment. We take the same approach as literature [35], in which we assign different numbers to the cells according to the relative positional relationship between cells, and then feed the these fixed positions with the serial numbers into the convolutional neural network.
  This location information is used as the environment for leader cell and follower cell, in which DQN is used to train cells. During the training process, the cell takes action according to strategy and selects one of the eight directions. Leader cell and follower cell will take as much action as possible to increase cumulative rewards, but their set of reward function are different that will be explained next.
- *Reward Function:* For the leading cell, its reward is similar to the setting method of the aforementioned paper, and is proportional to the Euclidean distance of the target position. The difference is that following the arrangement of the cells, in addition to considering the distance from the target cells, it is also necessary to use the stimulation signal as a function of suppression or acceleration. Leader cell moves according to the optimal motion trajectory, training follower cells to maintain the same direction and speed as the leader cell. The follower receives the stimulus signal from the leader, which speeds up the movement of the follower. If follower cell doesn’t match leader cell, the environment will give it a penalty.

## Experiments and results

Regarding the influence of stimulation signals on the migration of collective cells, this paper focuses on two sets of experiments to focus on, which proves that appropriate stimulation signals and release time of stimulation signals can have a huge impact on the migration rate of collective cells. The experimental environment of the cells is similar to the literature [35] and the cell morphology data is also referred to in this literature. We mainly want to compare the effects of stimulating signals and no stimuli on the simulation platform for the movement speed of collective cells. However, due to limited equipment and resources, we did not verify in the real cell scene. As the cell growing exponentially, the calculation time increases as it is positively correlated.

In the first set of experiments, there was only one recipient of the stimuli signal released by the leading cells. The difference is that in the second set of experiments, there were two recipients of the stimulus signal released by the leading cells. Therefore, when there are two recipients, it is also necessary to consider the competition and cooperation between the two agents for resources, because the two agents do not necessarily receive as many signals. The same is true for the two sets of experiments, during training the behavior policy was *ε*-greedy with *ε* gradually increasing from 0.35 to 0.9 and then remained unchanged. In all experiments, we trained the agent 3000 epochs to ensure that the experimental results were more fitting. After training, the action-value function of the network prediction has stabilized. Of course, in the process of training, each experience will be stored in the experience replay pool, after which each experience will be randomly selected from the experience pool for later training.

Since the experimental comparison results are also carried out on the simulation platform, in order to ensure the rationality of the experimental comparison, the setting of the reward function is different, and it is necessary to ensure the consistency of other hyperparameters.

### One leader cell and one follower cell

**Fig. 2:**
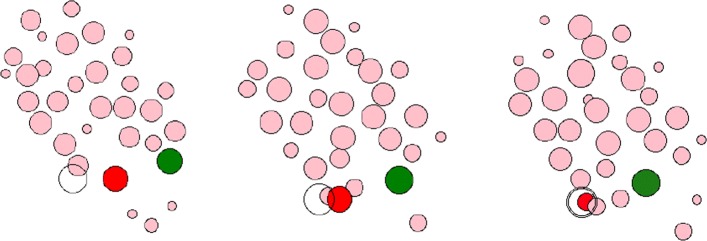
The movement process of a leader cell and a follower cell. Among them, the red ellipse represents the leader cell, the green ellipses represents the follower cell, the black open circle represents the target of the leader cell, and the remaining cells represent the neighboring cells during the movement. The relative positional relationship can be seen as environment of agent

Figure 2 shows how the following cell moves with the leading cell if there is only one recipient of the stimulus signal released. It can be seen that as the leading cell move over time, they gradually move toward the target direction until they reach the target position, and a cycle of the entire simulation ends. During a single simulation cycle, the following cell is always at a safe distance from the leading cell and are also moving toward the same target position as the leading cell. During the entire movement, the morphology and fate of other neighboring cells will also be considered, including the basic processes of cell division, growth, reproduction, and movement. But whether neighbor cells will also move toward the target direction of the leading cells is ignored in this experiment.

We use the relative positional relationship between cells and cells as well as the state of the cells as input to the neural network. The DQN algorithm is used to train the leading cells to obtain its optimal motion trajectory, and the motion trajectory after it is fixed as the optimal trajectory. The $P_{l}^{t_{0}}$ was used to indicate the starting center position of leading cell at time *t*_0_, $P_{f}^{t_{0}}$ to indicate follower’s, and the $P_{Target}^{{_{t}}_{0}}$ was represented as the target position.

4 $$ D_{t_{0}}^{L} = \left| P_{f}^{t_{0}} - P_{l}^{t_{0}}\right|  $$

Therefore, Eq. (4) represents the starting distance at time *t*_0_ between leader cell and follower cell, Eq. (5) represents the distance between follower and target.

5 $$ D_{t_{0}}^{T} = \left| P_{f}^{t_{0}} - P_{Target}^{{_{t}}_{0}}\right|  $$

When the leading cell does not release a stimulus signal to follow the cell, only use the change of $D_{t}^{T}\phantom {\dot {i}\!}$ between the next time $t^{'}\phantom {\dot {i}\!}$ and the current time *t* to set rewards. If $D_{t}^{T}$ remains the same or gets smaller, it indicates that it is moving in the direction of target cell, so the environment will give it a positive reward, on the contrary, it gives a negative punishment.

If the leading cell releases a stimulating factor to the following cell, then the reward function be related to the release of the stimuli signal. We used *I*_*t*_ to represent the interval of release signal, it will obtain reward based on $D_{t}^{T}$ and $D_{t_{0}}^{L}$, not just $D_{t}^{T}$. If $D_{t}^{T}$ and $D_{t_{0}}^{L}$ start reducing or unchanging, the action-value will change accordingly. Because in the training process of the neural network, small changes will bring great fluctuations.

**Fig. 3:**
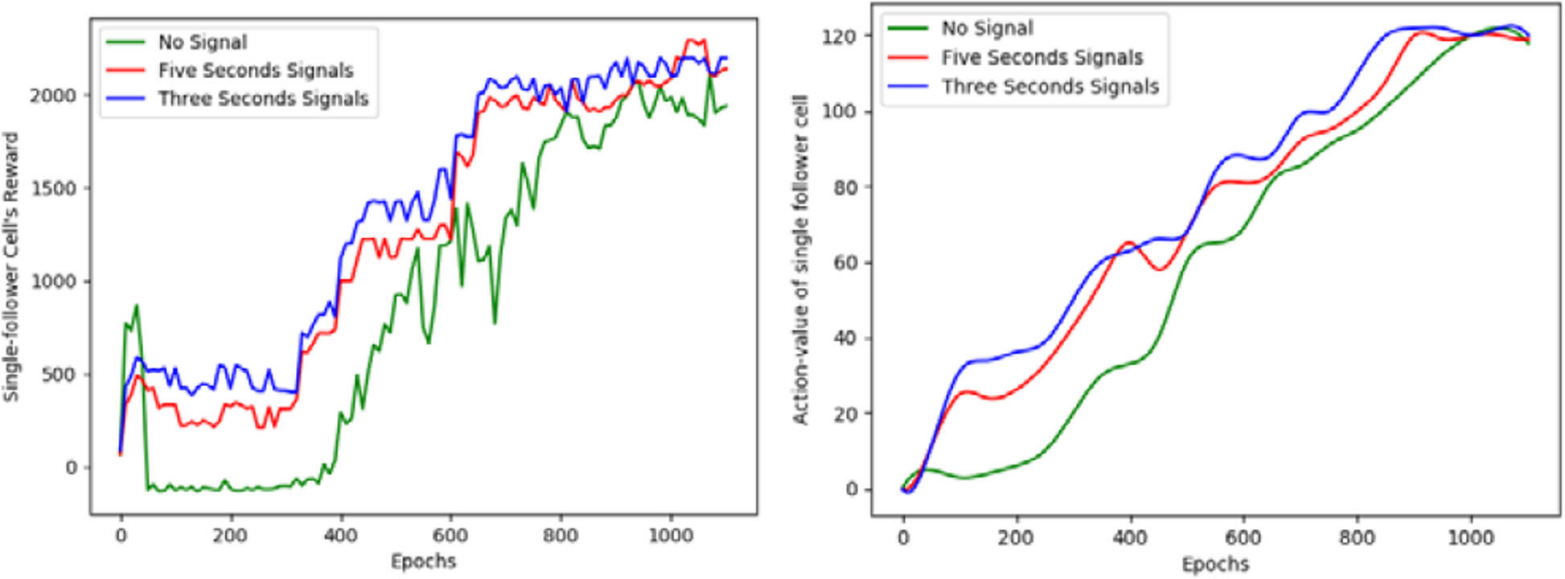
A quantitative behavioral representation of single follower cell. On the left, the abscissa is represented as the number of training, and the ordinate is expressed as the total rewards obtained by the agent. On the right side, the abscissa is also expressed as the number of training, and the ordinate represents the action-value of the agent. Among them, the green curve indicates that no stimulus signal is received, the red representative receives a stimulation signal every five seconds (*I*_*t*_=5*s*), and the blue color indicates that the stimulation signal is received every three seconds(*I*_*t*_=3*s*)

It can be seen from the contrast effect of the experiment that the following cell subjected to the stimulation signal are more efficient and more likely to converge than the cells without the stimulation signal. Figure 3 describes the evolution of the quantitative descriptors of behavior during training. From Fig. 3 we can also see that *I*=3*s* is faster to learn than *I*=5*s*, therefore with increasing the frequency of stimulation signals agent also learn faster.

### One leader cell and multi-follower cells

**Fig. 4:**
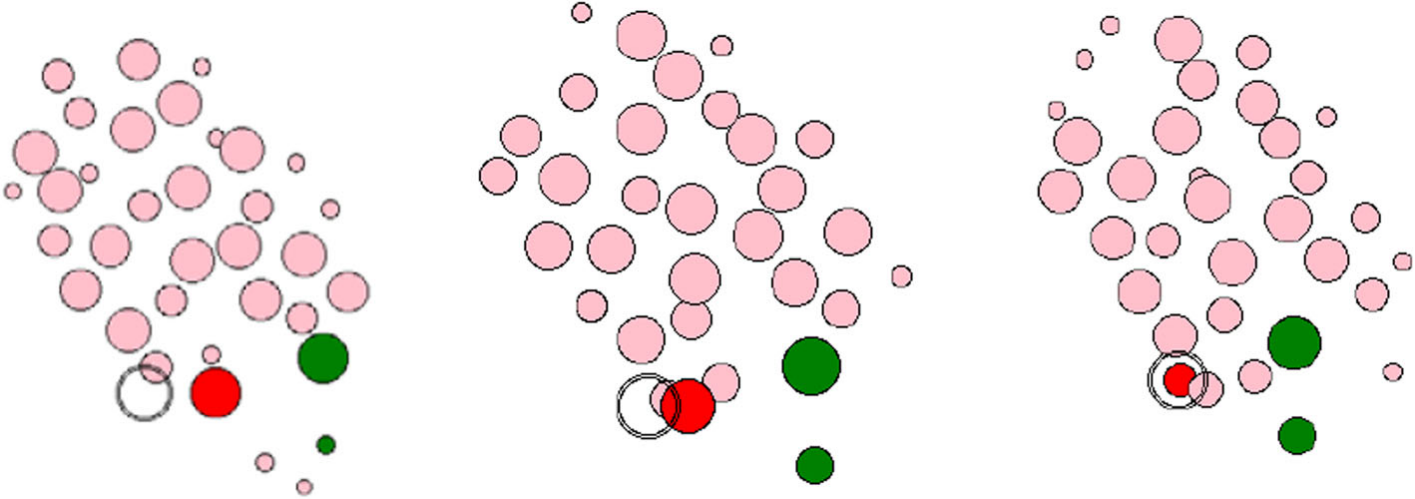
The movement process of a leader cell and multi-follower cells. The representation of the color is the same as the process of a single follower cell. The difference is that there are two follower cells here

When we increase the number of following cells to two, the moving process of multi-follower cells followed the leader cell to is shown in Fig. 4. The meaning of the ellipse color is the same with Fig. 2. As the leader cell moves toward the target, the two follower cells that maintain an appropriate distance from the leader cell are also moving toward the target.

The method of training multi-follower cells and leader cell are also consistent with the first set of experiments. We use $P_{f_{1}}^{t_{0}}$, $P_{f_{2}}^{t_{0}}$ to respectively indicate the starting center position of two follower cells at time *t*_0_. Follower cell-1 and follower cell-2 also use similar approach to calculate the distance just like the first experiment. We need to replace $P_{f}^{t_{0}}$ in Eqs. 4 and 5 with $P_{f_{1}}^{t_{0}}$ and $P_{f_{2}}^{t_{0}}$,respectively.

**Fig. 5:**
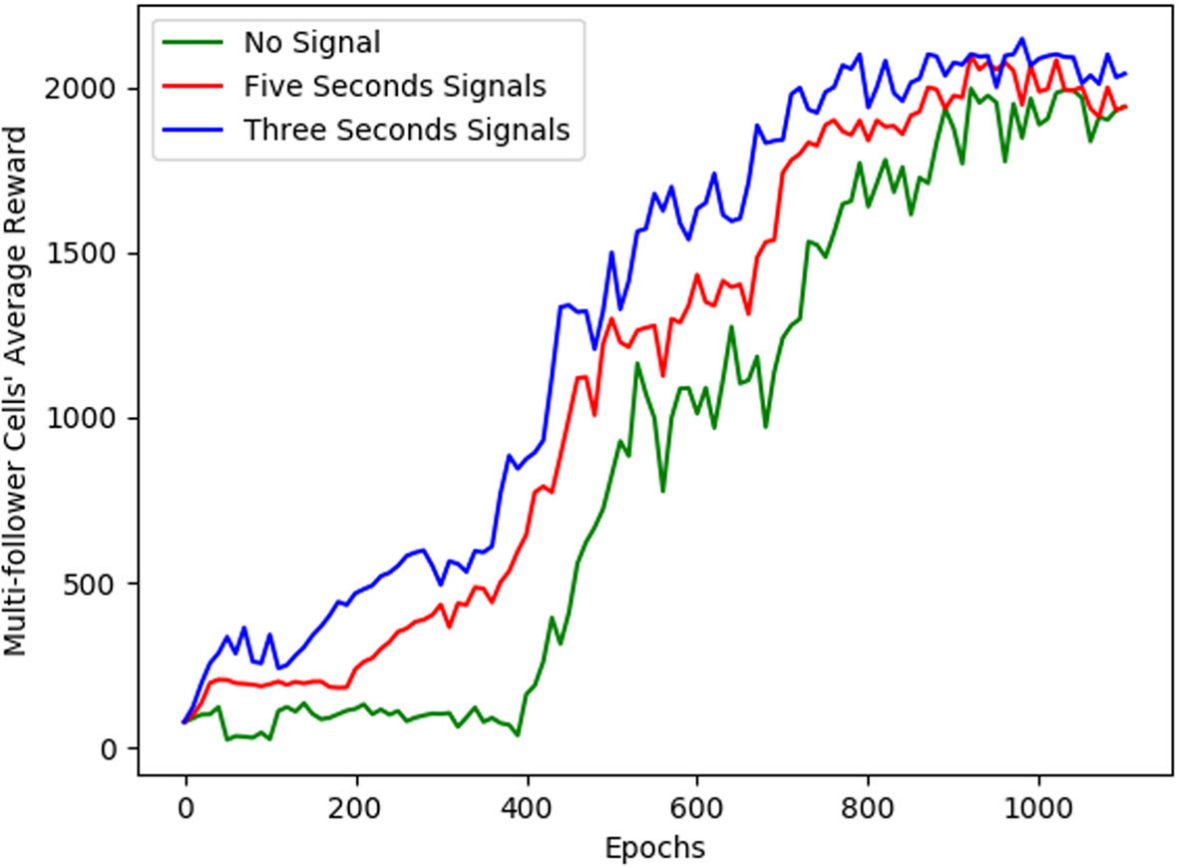
A quantitative behavioral representation of multi-follower cells. The representation of the abscissa is the same as single-follower cell, but the ordinate is calculated as the average of the total rewards

In the process of multi-following cells, we do not consider the phenomenon of competition and cooperation between them, so the training process for multiple followers and single followers is the same. As shown in Fig. 5, for multiple followers, the stimulation signal still increases its learning speed. So we can get that the number of stimulation signals is related to the learning speed, and there is a proportional relationship between them. However, the leader cell must consider two follower cells simultaneously in multi-follower. The agent that receiving the stimulation signals requires an average of 210 steps, while agents that do not receive the stimulation signals require an average of more steps, which typically require 222 steps.

## Discussion

Accelerating the speed of collective cell migration can play a important role in many biological areas, such as organizational development, wound healing, cancer metastasis, and immune response. By studying the effect of stimulation signals on the moving speed of follower cell, we get the conclusion that stimulation signals can control the movement of follower cells, thereby accelerating the rate of collective cell migration.But due to the truth that there is no mature way to control the signal release from the leader cell in the real scene, so we can not effectively use the real cell migration scenario to do experiments, and just do it in simulation environment.

There are some aspects can be improved in the experiment: (1) First of all, in the reward setting function of our experiment, a fixed number stimulation signals were released to the follower cell at equal time interval.In the future, the leader cell can be trained to learn to control the signal interval so that it can automatically release signals instead of manually setting the same time interval. (2) Second, the interaction between the leader and the follower only obtains the location information of each other in this experiment.But there are other informations in the cells, which may effect the state. So we can make the follower and leader cells receive more informations such as the direction and speed about the current state in the future. (3) Third, we study the leader cells and the follower cells which will move toward the target direction, while other cells which may also move toward the same target are ignored. In the actual scene the resources shared by the cells are limited, so there will be competition and cooperation between the cell which move toward the same direction.This phenomenon of competition and cooperation can also continue to be studied in depth in future work.

## Conclusions

For the algorithm itself in the simulation environment, this paper is based on a simple DQN. Therefore, in the following work, we can also concentrate more on improving the current algorithms. In addition, this paper only considers the relationship between leader cells and follower cells, but in fact the relationship between cells is considerable complexes, and the follower cells are also stimulated and acted upon by other cells.
